# Exploring the Cyclic Patterns of Secondary Hair Follicles in Cashmere Goats Based on Skin Transcriptome Data

**DOI:** 10.3390/ani16142156

**Published:** 2026-07-11

**Authors:** Gao Gong, Yuekun Tang, Jianqing Zhao, Mengting Zhu, Aladaer Qi, Shijie Bi, Yiming Sulaiman, Wenxin Zheng

**Affiliations:** 1 College of Animal Science, Xinjiang Agricultural University, Urumqi 830052, China; 15621219363@163.com (Y.T.); zhao2021@nwafu.edu.cn (J.Z.); zhumengting@xjau.edu.cn (M.Z.); aqi@xjau.edu.cn (A.Q.); ysulaiman@xjau.edu.cn (Y.S.); 2 College of Food Science and Pharmacy, Xinjiang Agricultural University, Urumqi 830052, China; bsj19941001@163.com

**Keywords:** cashmere goat, RNA-seq, secondary hair follicle cycle, regulatory genes

## Abstract

The cashmere fibers produced by the secondary hair follicles of cashmere goats are precious textile raw materials. The secondary hair follicles exhibit a distinct annual cyclical pattern, comprising anagen, catagen, and telogen phases. Although a large amount of skin transcriptome data is currently available for cashmere goats, this study aims to systematically investigate the regulatory factors controlling the cyclical dynamics of secondary hair follicles through re-analysis of these data. Skin transcriptome data of Inner Mongolian cashmere goats were downloaded from the Sequence Read Archive (SRA) database. Bioinformatics analyses, including quality control, read mapping, quantification, differential expression analysis, Gene Ontology (GO) enrichment analysis, and KEGG pathway analysis, were performed to identify key regulatory genes governing the secondary hair follicle cycle. The results revealed a total of 1232 differentially expressed genes (DEGs) from comparisons across different phases. KEGG pathway analysis identified three signaling pathways associated with cashmere development: the cAMP signaling pathway (chx04024), the relaxin signaling pathway (chx04926), and the estrogen signaling pathway (chx04915). GO analysis yielded 335 terms, of which 236 were statistically significant (*P-adj* < 0.05). The DEGs were predominantly involved in biological processes such as regulation of transcription by RNA polymerase II, proteolysis, and positive regulation of transcription by RNA polymerase II. Significantly enriched cellular components included the plasma membrane, membrane, and extracellular space, and molecular functions were mainly related to protein binding, identical protein binding, and DNA-binding transcription factor activity, RNA polymerase II-specific. Based on their expression patterns, five key genes were selected: *KRT25*, *KRT39*, *MAPK12*, *SPP1*, and *TCHHL1*. These genes displayed significantly distinct expression profiles across the hair follicle cycle. *TCHHL1* exhibited the highest expression in anagen, intermediate expression in catagen, and the lowest expression in telogen, with significant differences among all three phases. *MAPK12* was specifically upregulated in telogen and was significantly more highly expressed than in both anagen and catagen. *SPP1* showed high expression in anagen and catagen but extremely low expression in telogen. *KRT39* displayed significantly higher expression in both anagen and catagen compared to telogen. *KRT25* reached its highest expression in catagen, which was significantly different from that in telogen, while its expression in anagen showed no significant difference from either telogen or catagen. This study characterized the regulatory genes of the secondary hair follicle cycle in cashmere goats at the transcriptional level and analyzed the association between their expression patterns and cycle regulation.

## Simple Summary

Cashmere fibers from secondary hair follicles are valuable textile materials, and these follicles undergo an annual cycle (anagen, catagen, and telogen). To systematically investigate regulatory factors, we re-analyzed skin transcriptome data of Inner Mongolian cashmere goats from the SRA database. Bioinformatics analyses (quality control, mapping, quantification, differential expression, GO, and KEGG) identified 1232 differentially expressed genes (DEGs) across phases. KEGG enriched three pathways: cAMP, relaxin, and estrogen signaling. GO analysis yielded 335 terms, involving transcription regulation, proteolysis, membrane components, and protein binding. Five key genes—*KRT25*, *KRT39*, *MAPK12*, *SPP1*, and *TCHHL1*—showed distinct expression patterns. Notably, *TCHHL1* peaked in anagen and was lowest in telogen; *MAPK12* was specifically high in telogen; *SPP1* was high in anagen/catagen but low in telogen; *KRT39* was high in anagen/catagen vs. telogen; and *KRT25* was highest in catagen. These expression profiles suggest their potential roles in hair cycle regulation. This study characterizes transcriptional regulators of the secondary hair follicle cycle and their association with phase transitions.

## 1. Introduction

The cashmere produced by cashmere goats is of superior quality. As a precious high-grade textile material, cashmere is characterized by its fineness, softness, light weight, warmth, and soft luster and has long been renowned as “soft gold” [1,2,3]. Cashmere goats are widely distributed across China, with abundant genetic resources. Different breeds exhibit significant differences in cashmere performance and adaptability, and a number of superior breeds with distinctive regional characteristics have been developed. The secondary hair follicles of cashmere goats produce cashmere fibers, and the growth and shedding of cashmere follow a pronounced annual cyclical pattern, which is clearly divided into the anagen, catagen, and telogen phases. In Inner Mongolian cashmere goats, the anagen phase of secondary hair follicles lasts from April to November, the catagen phase occurs in December and January, and the telogen phase spans February and March. Among these, the anagen phase is the critical regulatory period determining cashmere yield and fiber length.

Currently, a large number of studies have focused on exploring the cyclical transition of secondary hair follicles. Research has demonstrated that photoperiod, nutritional level, hormones, various signaling pathways, and key genes are all involved in the regulation of the secondary hair follicle cycle [4,5,6,7], with hormones, signaling pathways, and key genes widely recognized as the two major categories of core factors affecting the cyclical regulation of secondary hair follicles [4,6,8]. Early RNA-sequencing studies in cashmere goats similarly revealed hundreds of differentially expressed genes between anagen, catagen, and telogen, with significant enrichment in Wnt, Shh, TGF-β, and Notch signaling pathways [9]. Duan et al. [6] found that photoperiod not only drives the synthesis of melatonin in the pineal gland, but exogenous implantation of melatonin can also advance the initiation of the anagen phase of secondary hair follicles and prolong its duration, increasing cashmere yield by 34.5% and fiber length by 21.3%. Meanwhile, photoperiod itself exerts a direct regulatory effect: short-day photoperiod promotes the entry of secondary hair follicles into anagen, whereas long-day photoperiod induces their transition into catagen and telogen [4,10]. Nutritional level can also affect the secondary hair follicle cycle: At the level of signaling pathways and genes, Wang et al. [8] confirmed that the Wnt/β-catenin pathway plays a critical regulatory role in the hair follicle cycle. It is highly activated during anagen and can regulate the proliferation and differentiation of stem cells and dermal papilla cells, thereby affecting hair shaft growth. Regarding this pathway, Gong et al. [11] found that overexpression of Wnt10b, an upstream ligand of the pathway, significantly enhances the proliferative capacity of dermal papilla cells; Su et al. [12] indicated that FGF5 is highly expressed in catagen and inhibits hair follicle growth, with its expression pattern exhibiting crosstalk with the Wnt/β-catenin pathway; and Wang et al. [8] showed that *LHX2*, a transcription factor that maintains the properties of hair follicle stem cells, exhibits elevated expression during anagen and can synergistically regulate stem cell activity with Wnt signaling.

At present, investigations into the cyclical patterns of secondary hair follicles in cashmere goats are primarily conducted at the transcriptomic level. Yang et al. [4] utilized RNA-seq technology to delineate the molecular classification of the anagen, catagen, and telogen phases of secondary hair follicles through differential expression analysis, functional enrichment, and clustering methods and identified March, September, and December as critical time points for phase transition. Time-series transcriptomic analysis revealed a pattern of initial activation followed by subsequent inhibition of the Wnt signaling pathway [13]. By integrating weighted gene co-expression network analysis (WGCNA) with proteomics, Han et al. [5] identified ADAM17, SFRP1, and PPP1CA as three core proteins regulating the transition of secondary hair follicles from telogen to anagen in cashmere goats. A large amount of skin transcriptome data on the cyclical patterns of secondary hair follicles in cashmere goats has been accumulated in public databases; however, key genes that can definitively regulate the cycle have yet to be identified. This study aims to download the skin transcriptome data of Inner Mongolian cashmere goats and conduct a more in-depth investigation into the cyclical growth of secondary hair follicles from the dimensions of large samples and multiple populations, with the goal of discovering novel molecular markers for the secondary hair follicle cycle in cashmere goats.

## 2. Materials and Methods

### 2.1. Data Sources

By searching the NCBI SRA database using the keywords “Inner Mongolian cashmere goat” and “skin”, a total of 132 skin transcriptome datasets of Inner Mongolian cashmere goats were collected and downloaded. Detailed information on the downloaded data is provided in Supplemental Table S1. These data were derived from four BioProjects: PRJNA470971, PRJNA382893, PRJNA592400, and PRJNA832904 (Table 1). All samples were skin transcriptomes from female Inner Mongolian cashmere goats. Based on sampling month and previously reported seasonal changes in secondary hair follicle activity, the transcriptome samples were classified into the anagen (April–November), catagen (December and January), and telogen (February and March) phases. This classification yielded 90 anagen, 21 catagen, and 21 telogen samples. Although the sample numbers were not fully balanced across BioProjects, all three phases were represented in three independent BioProjects. These samples were collected at different time points and from different sampling populations, which enabled us to integrate transcriptomic data from multiple sources and identify common candidate gene markers potentially involved in the transition of the secondary hair follicle cycle. The transcriptome data were then renumbered as IMCG_c01 (catagen), IMCG_t01 (telogen), and IMCG_a01 (anagen).

### 2.2. Data Quality Control

The downloaded transcriptome reads were trimmed using Trimmomatic software (version 0.39) [7] for sequence quality control. All downloaded RNA-seq datasets were processed as paired-end sequencing data. The parameters applied were ILLUMINACLIP:TruSeq3-PE.fa:2:30:10, LEADING:3, TRAILING:3, SLIDINGWINDOW:4:15, and MINLEN:36.

### 2.3. Alignment to the Reference Sequence

In this study, the quality-controlled and filtered sequencing data were aligned to the goat genome (assembly version: GCF_001704415.2_ARS1) using HISAT2 (version 2.2.1) [14]. To improve alignment accuracy and efficiency, the following parameters were set: -p 60, which specifies the use of 60 threads for parallel computation to ensure rapid processing; --dta, an option that optimizes the alignment output format for better compatibility with downstream transcript assemblers; --no-unal, which suppresses the output of unaligned sequences to avoid generating redundant data; and --un-conc-gz was used to output paired-end reads that failed to align concordantly in gzip-compressed format.

### 2.4. Expression Quantification

In this study, gene expression quantification was performed on skin tissue samples from Inner Mongolian cashmere goats using StringTie software (version 2.2.1) [15]. Several parameters were set during the analysis to optimize the results: the -e parameter, which restricts expression estimation to the reference genome annotation and efficiently estimates the expression levels of known transcripts; the -B parameter, which ensures that the output files are compatible with subsequent differential expression analysis; the -p 50 parameter, which substantially improves computational efficiency by invoking 50 threads. Gene expression quantification was performed using StringTie. Gene expression quantification was performed using StringTie. After quantification, the gene-level raw count matrix was obtained using prepDE.py and was used for downstream differential expression analysis. The FPKM matrix was obtained using getPFKM.py and was used only for visualization of gene expression distribution and expression pattern analysis.

### 2.5. RNA-Seq Differential Expression Analysis

After the mRNA expression matrix data were obtained, they were analyzed using R (version 4.3.3), RStudio (version 2024.9.1.394), and other software. Principal component analysis (PCA), differential expression analysis, KEGG pathway analysis, GO functional enrichment analysis, and screening of key genes were conducted to identify the key genes and signaling pathways influencing the anagen, catagen, and telogen phases of secondary hair follicles in Inner Mongolian cashmere goats.

#### 2.5.1. Differential Expression Analysis

Differential expression analysis was performed using the DESeq2 package (version 1.46.0) [16]. The input matrix for DESeq2 consisted of raw read counts for each gene across all samples. The design formula used in DESeq2 was design = ~group, where “group” represented the secondary hair follicle cycle phase, including anagen, catagen, and telogen. BioProject or batch information was not included as a covariate in the differential expression model. The thresholds for significant differential expression were set as |log_2_FC| > 1 and adjusted *p*-value (*P-adj*) < 0.01. Volcano plots of differentially expressed genes were generated using the R package ggplot2 (version 3.5.2), and Venn diagrams were constructed using the online tool Jvenn with reference to the tutorial [17] at https://www.bioinformatics.com.cn/static/others/jvenn/example.html (accessed on 8 July 2026).

#### 2.5.2. KEGG Pathway and GO Functional Enrichment Analysis of Differentially Expressed Genes

In this study, the Gene Ontology (GO) method was used to annotate and classify gene functions based on three categories: Cellular Component (CC), Biological Process (BP), and Molecular Function (MF). The functions of the differentially expressed genes were then analyzed, which, in combination with the research objectives, can help identify relevant functions and genes [18,19]. For pathway enrichment analysis, this study utilized the Kyoto Encyclopedia of Genes and Genomes (KEGG) database. The differentially expressed genes identified from the Inner Mongolian cashmere goat transcriptome were analyzed using their gene IDs as input to identify the associated pathways. Simultaneously, GO functional enrichment analysis and KEGG pathway enrichment analysis were performed using DAVID software (version 2025) [20]. Here, *P-adj* represents the adjusted *p*-value, and a term or pathway was considered significantly enriched when *P-adj* < 0.05. Finally, GO classification charts and KEGG scatter plots were generated for the enriched terms to provide an intuitive visualization of the analysis results. Protein–protein interaction (PPI) networks were constructed for genes enriched in selected KEGG pathways using the STRING database, with Capra hircus selected as the organism. Interactions were retrieved using a medium-confidence score threshold. In the PPI networks, nodes represent proteins and edges represent predicted or known protein–protein associations.

### 2.6. Screening of Candidate Stage-Associated Genes

Candidate stage-associated genes were screened based on the integrated results of differential expression analysis, GO functional enrichment, KEGG pathway enrichment, expression pattern analysis, and previous literature. First, genes were required to be identified as differentially expressed genes (DEGs) in at least one pairwise comparison among the anagen, catagen, and telogen phases, using the thresholds |log_2_FC| > 1 and adjusted *p*-value < 0.01. Second, genes showing distinct phase-associated expression patterns across the three secondary hair follicle cycle phases were prioritized. Third, genes associated with enriched GO terms or KEGG pathways related to hair follicle development, keratinization, extracellular microenvironment remodeling, immune response, or signaling regulation were further considered. Fourth, genes previously reported to be involved in skin development, hair follicle biology, keratinocyte differentiation, extracellular matrix remodeling, or related biological processes were given priority. Finally, genes with clear expression differences among phases and suitable primer design for qRT-PCR validation were selected for experimental verification. Based on these criteria, *KRT25*, *KRT39*, *SPP1*, *TCHHL1*, and *MAPK12* were selected as candidate stage-associated genes for subsequent qRT-PCR validation. These genes were considered putative regulatory candidates associated with the secondary hair follicle cycle rather than genes with experimentally confirmed regulatory functions.

### 2.7. Quantitative Real-Time PCR (qRT-PCR)

Specific primers for quantitative real-time PCR were designed using Primer-BLAST (version 2.17.0) in the NCBI database based on the mRNA coding sequences of the goat *KRT25*, *KRT39*, *TCHHL1*, *SPP1*, *MAPK12*, *GAPDH*, and *β-actin* genes, and all primers were synthesized by Sangon Biotech (Shanghai) Co., Ltd. (Shanghai, China) (primer sequences are listed in Table 2). qRT-PCR was performed using the TB Green-based qRT-PCR kit from TaKaRa Biotechnology (Beijing, China). The experimental samples consisted of skin tissue cDNA from adult female Inner Mongolian cashmere goats, including 10 samples each from the anagen (September), catagen (December), and telogen (March) phases. Each qRT-PCR reaction was performed in technical triplicate for each biological sample. For each sample, the geometric mean of the Ct values of *GAPDH* and *β-actin* was used as the reference Ct value for normalization in the 2^−ΔΔCt^ calculation.

Statistical analysis of qRT-PCR relative expression data was performed using R. According to the secondary hair follicle cycle phase, the samples were divided into three groups: anagen, catagen, and telogen. For each gene, relative expression levels among the three phases were analyzed by one-way analysis of variance (ANOVA), followed by Duncan’s multiple-range test using the agricolae package (version 1.3-7). The tidyverse package (version 2.0.0) was used for data organization and format conversion, and ggplot2 and patchwork (version 1.3.2) were used for visualization. Bar charts were generated to show the mean ± SEM of relative expression levels, and different letters above the bars indicate highly significant differences among phases (*p* < 0.01).

## 3. Results

### 3.1. Reference Genome Alignment Analysis

The results of the reference genome alignment analysis are presented in Supplemental Table S2. Across all 132 samples, the total number of sequencing reads (total_reads) ranged from 3,172,620 to 53,132,241, with a mean of 19,818,431 reads and a median of 23,857,696 reads, indicating variation in sequencing depth among samples. In terms of alignment efficiency, the unique mapping rate (unique_map) ranged from 68.81% to 90.67%, with a mean of 82.98% and a median of 81.38%. The overall mapping rate (total_map) ranged from 85.58% to 98.65%, indicating that the alignment quality was generally satisfactory for subsequent transcriptomic analysis.

Principal component analysis (PCA) was performed based on the transcriptome gene expression matrix to evaluate the overall distribution of samples (Supplemental Figure S1). PC1 and PC2 explained 24.9% and 15.6% of the total variance, respectively. The PCA results showed that samples from the three secondary hair follicle cycle phases could be broadly distinguished, suggesting that the transcriptome profiles captured phase-associated expression differences among anagen, catagen, and telogen samples.

### 3.2. RNA-Seq Dataset Analysis

**Figure 1:**
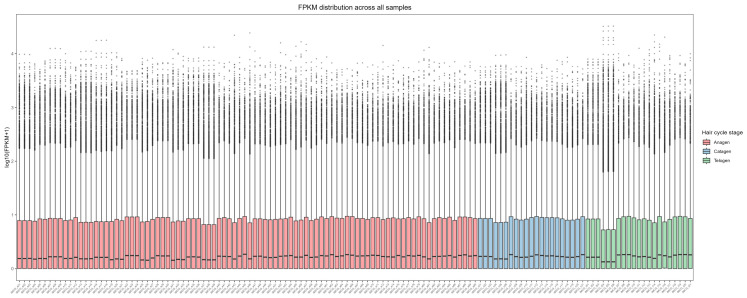
Distribution of gene FPKM across all samples.

The expression distribution of FPKM values was analyzed for each sample. A violin plot of gene FPKM values was generated for all samples (Figure 1), which revealed that the distribution pattern and magnitude of the log_10_(FPKM + 1) values were largely consistent across all groups.

### 3.3. Differentially Expressed Gene Analysis

**Figure 2:**
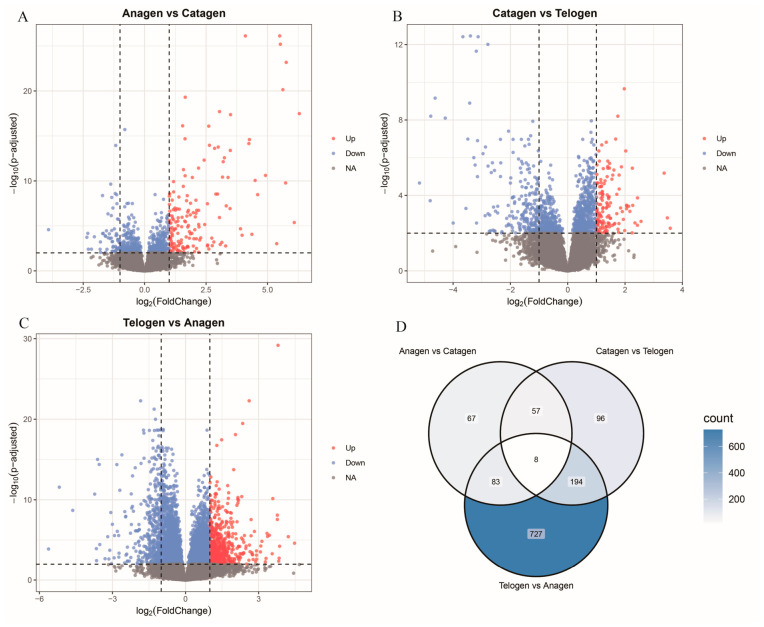
Volcano plots and Venn diagram of differentially expressed genes across secondary hair follicle cycles in Inner Mongolian cashmere goats. Panels (**A**–**C**) show volcano plots of differentially expressed mRNAs in cashmere goats at different cycle stages. The x-axis represents log_2_(fold change). The y-axis represents −log_10_ (*P-adj*), indicating statistical significance (*P-adj* < 0.01 as the threshold). Panel (**D**) shows the Venn diagram.

Pairwise differential expression analyses were performed among the three phases of the secondary hair follicle cycle (anagen vs. catagen, anagen vs. telogen, and catagen vs. telogen) using skin transcriptome data from Inner Mongolian cashmere goats. A total of 1232 unique differentially expressed genes (DEGs) were identified after merging the results from the three comparisons and removing duplicates (|log_2_FC| > 1, adjusted *p*-value < 0.01). The statistical summary is provided in Table 3. In the individual comparisons, 215 DEGs were detected in anagen vs. catagen (157 upregulated, 58 downregulated), 1012 DEGs in anagen vs. telogen (611 upregulated, 401 downregulated), and 355 DEGs in catagen vs. telogen (139 upregulated, 216 downregulated). Eight genes (*KRT4*, *BPIFB2*, *LOC102191166*, *LOC106503120*, *LOC102185848*, *LOC102188576*, *LOC108633210*, and *LOC108634363*) were common to all three comparisons. Volcano plots and a Venn diagram illustrating these results are shown in Figure 2, and detailed information on all DEGs is provided in Supplementary Table S3.

### 3.4. Enrichment Analysis of Differentially Expressed Genes

Based on the 1232 differentially expressed genes (DEGs) identified across the three secondary hair follicle cycle phases, GO functional annotation and KEGG pathway enrichment analyses were performed. A total of 335 GO terms were enriched, of which 236 were significant (*P-adj* < 0.05), and the detailed GO enrichment results are provided in Supplemental Table S4. Among the three GO categories, 67 Molecular Function (MF) terms were enriched, including 51 significant terms, mainly involving protein binding, identical protein binding, DNA-binding transcription factor activity, structural molecule activity, calcium ion binding, and steroid binding. A total of 214 Biological Process (BP) terms were enriched, including 143 significant terms, mainly associated with regulation of transcription by RNA polymerase II, proteolysis, cell differentiation, transmembrane transport, neutrophil chemotaxis, lymphocyte chemotaxis, and chemokine-mediated signaling pathways. In addition, 54 Cellular Component (CC) terms were enriched, including 42 significant terms, mainly related to the plasma membrane, membrane, extracellular space, extracellular region, cytosol, and keratin filament. Further analysis of the GO classification chart showed that several BP terms converged on proteolysis, multiple CC terms were associated with extracellular space, and several MF terms were related to calcium ion binding, suggesting that protein degradation, extracellular microenvironment remodeling, and ion-binding functions may be involved in the regulation of the secondary hair follicle cycle in Inner Mongolian cashmere goats.

The KEGG pathway enrichment analysis showed that 428 genes were enriched in KEGG pathways, with a total of 31 pathways enriched and 22 of them significantly enriched. The KEGG enrichment results are presented in Supplemental Table S5. The DEGs were mainly enriched in signaling pathways such as cornified envelope formation, neuroactive ligand–receptor interaction, estrogen signaling pathway, and cAMP signaling pathway.

**Figure 3:**
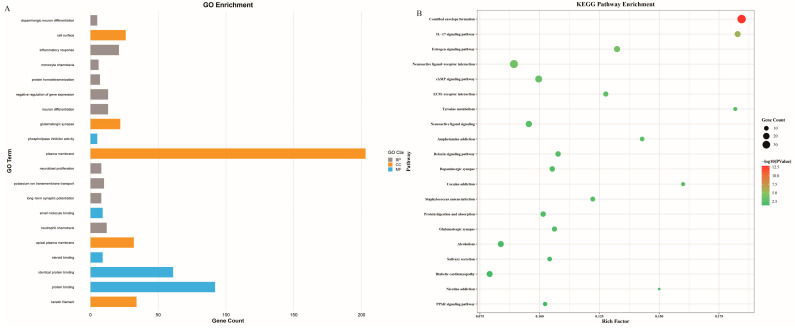
Enrichment analysis plot of differentially expressed genes: (**A**) Bar chart of GO enrichment analysis of differentially expressed genes. (**B**) Bubble plot of KEGG enrichment analysis of differentially expressed genes.

Further analysis showed that the DEGs were predominantly enriched in cellular components such as extracellular space, keratin filament, and extracellular region. The associated molecular functions mainly included protein binding, calcium ion binding, and structural molecule activity. The biological processes involved were intermediate filament organization, epithelial cell differentiation, transmembrane transport, and chemokine-mediated signaling pathways. KEGG pathway analysis indicated that the DEGs were mainly enriched in terms such as cornified envelope formation, neuroactive ligand–receptor interaction, cAMP signaling pathway, and estrogen signaling pathway. A bar chart was generated for representative significant GO terms (Figure 3A), and a bubble plot was generated for significant KEGG pathways (Figure 3B). These results suggest that these pathways may play crucial roles in regulating extracellular matrix organization, immune response, neurodevelopment, and keratinization-related processes. The significantly enriched KEGG pathways mainly included cornified envelope formation, estrogen signaling pathway, cAMP signaling pathway, and ECM–receptor interaction.

### 3.5. cAMP Signaling Pathway

**Figure 4:**
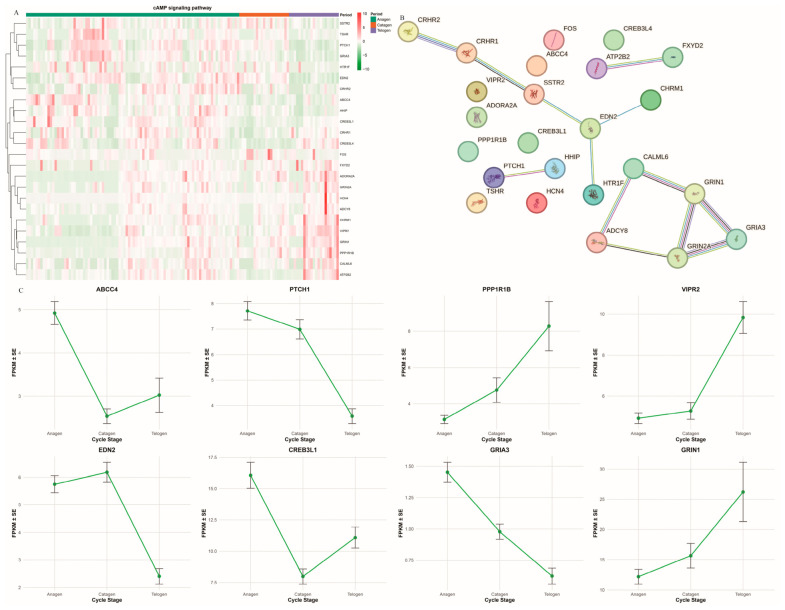
Expression analysis of genes in the cAMP signaling pathway: (**A**) clustering heatmap of all differentially expressed genes; (**B**) protein–protein interaction (PPI) network analysis; (**C**) expression trend diagram of selected key genes. In panel (**C**), the x-axis represents the anagen, catagen, and telogen stages (hair follicle cycle), and the y-axis represents RNA-seq FPKM values.

The cAMP signaling pathway is a key signaling pathway that promotes the maintenance of the anagen phase of secondary hair follicles and the activity of hair follicle stem cells in cashmere goats, playing an important role in the positive regulation of the hair follicle cycle. Analysis of the 24 genes enriched in this pathway was performed. The gene clustering heatmap (Figure 4A) revealed that *HCN4* and *ADCY8* exhibited relatively high expression levels in telogen, *CRHR2* and *CREB3L1* were highly expressed in anagen, and FOS showed high expression in catagen. Protein–protein interaction (PPI) network analysis of these genes (Figure 4B) identified direct interactions among some of the encoded proteins. Among them, the EDN2 protein was located at the center of the network, linking three protein networks, and a close network association was observed between CRHR1 and CRHR2. We further analyzed the gene expression trends for a subset of genes (Figure 4C). *PPP1R1B*, *VIPR2*, and *GRIN1* displayed consistent expression trends. In this pathway, their expression differed markedly between anagen and catagen.

### 3.6. Estrogen Signaling Pathway

**Figure 5:**
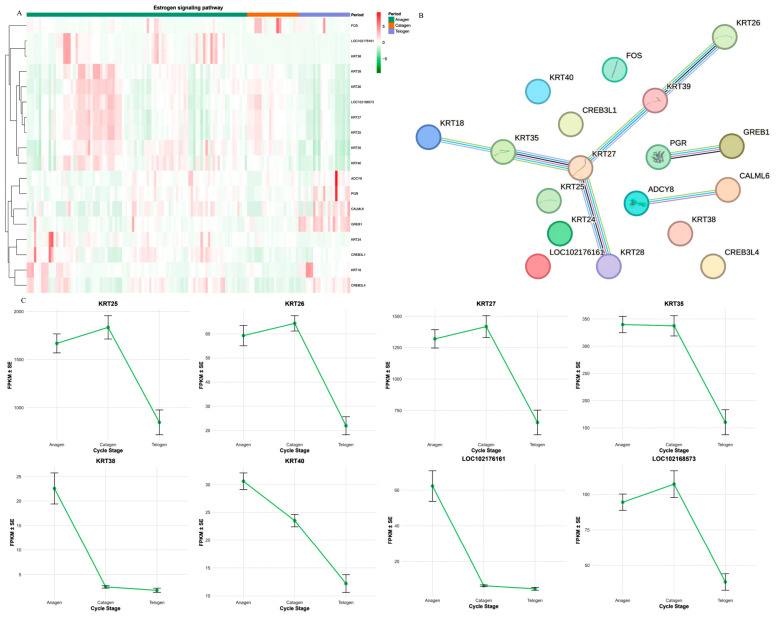
Analysis of gene expression in the estrogen signaling pathway: (**A**) clustering heatmap of all differentially expressed genes; (**B**) protein–protein interaction (PPI) network analysis; (**C**) expression trend diagram of selected key genes. In panel (**C**), the x-axis represents the anagen, catagen, and telogen stages (hair follicle cycle), and the y-axis represents RNA-seq FPKM values.

The estrogen signaling pathway is a key signaling pathway that regulates the initiation of the catagen phase of secondary hair follicles in cashmere goats and plays an important stage-specific regulatory role in hair follicle cycle transition. Analysis of the 18 genes enriched in this pathway was performed. The gene clustering heatmap (Figure 5A) revealed that *PGR* and *ADCY8* exhibited relatively high expression levels in telogen, *KRT24* and *CREB3L1* were highly expressed in anagen, and FOS showed high expression in catagen. Protein–protein interaction (PPI) network analysis of these genes (Figure 5B) identified direct interactions among some of the encoded proteins. Among them, the KRT27 protein was located at the center of the network, linking three protein networks, and a close network association was observed between KRT39 and KRT26. We further analyzed the gene expression trends for a subset of genes (Figure 5C). *KRT25*, *KRT26*, *KRT27*, and *KRT35* displayed consistent expression trends. In this pathway, their expression differed markedly between anagen and catagen, with the lowest levels observed in catagen for all these genes.

### 3.7. Pathway Network and Key Gene Analysis

**Figure 6:**
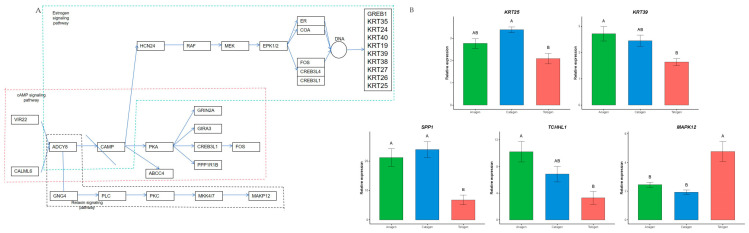
Bar charts of five significantly differentially expressed genes and the connections among the cAMP, relaxin, and estrogen signaling pathways: (**A**) connections among pathways, different colored dashed boxes represent different signaling pathways; (**B**) the y-axis represents qRT-PCR relative expression levels calculated using the 2^−ΔΔCt^ method. Different uppercase letters above the bars indicate extremely significant differences (*p* < 0.01), whereas the same letters indicate no significant difference.

Based on the analysis of key pathways and terms, combined with the growth and development of secondary hair follicles and previous studies, several key candidate genes were screened (Table 4). These genes were differentially expressed in the skin tissue of Inner Mongolian cashmere goats and also exhibited expression differences across different growth stages of the secondary hair follicles. Five key pathways were identified: the estrogen signaling pathway, cAMP signaling pathway, relaxin signaling pathway, cornified envelope formation, and ECM–receptor interaction. Among them, the estrogen, relaxin, and cAMP signaling pathways were interconnected. Based on the KEGG network, a KEGG molecular network of key genes in the secondary hair follicle cycle was constructed (Figure 6A).

Further statistical analysis of all enriched KEGG pathways revealed that the differentially expressed genes were predominantly enriched in the cAMP, relaxin, and estrogen signaling pathways. The interconnections among these three pathways and the expression bar charts of the five key genes are presented in Figure 6. Most of these signaling pathways are closely related to cashmere growth and development. The genes enriched in the cAMP signaling pathway included *CALML6*, *CREB3L1*, *ABCC4*, *PPP1R1B*, *GRIN2A*, and *GRIA3*; genes enriched in the relaxin signaling pathway included *MAPK12* and *PTCH1*; and genes enriched in the estrogen signaling pathway included *KRT18*, *KRT24*, *KRT25*, *KRT26*, *KRT27*, *KRT35*, *KRT38*, *KRT39*, *KRT40*, *CREB3L1*, *CREB3L4*, *FOS*, *ADCY8*, and *GREB1*.

Five candidate genes associated with the secondary hair follicle cycle, *KRT39*, *MAPK12*, *KRT25*, *SPP1*, and *TCHHL1*, were further selected for qRT-PCR validation (Figure 6B). The results showed that *KRT25* exhibited the highest expression level in catagen, with a significant difference compared with telogen (*p* < 0.01), whereas no significant differences were observed between anagen and telogen or between anagen and catagen. *KRT39* expression was significantly higher in anagen than in telogen (*p* < 0.01), while no significant differences were observed between catagen and anagen or between catagen and telogen. *SPP1* showed comparable expression levels in anagen and catagen, both of which were significantly higher than that in telogen (*p* < 0.01). *TCHHL1* expression was highest in anagen, intermediate in catagen, and lowest in telogen, with anagen expression being significantly higher than telogen expression (*p* < 0.01). *MAPK12* expression was significantly higher in telogen than in anagen and catagen (*p* < 0.01), whereas no significant difference was observed between anagen and catagen. These results indicate that *KRT25*, *KRT39*, *SPP1*, *TCHHL1*, and *MAPK12* exhibit phase-associated expression patterns during the secondary hair follicle cycle, suggesting that they may represent candidate genes involved in stage-specific changes of secondary hair follicles in cashmere goats.

## 4. Discussion

This study systematically analyzed key genes and regulatory pathways involved in the cyclical development of secondary hair follicles through integrative analysis of multiple skin transcriptome datasets from Inner Mongolian cashmere goats and identified cAMP, relaxin [21], and estrogen signaling pathways as important pathways associated with the hair follicle cycle. Furthermore, five key genes—*KRT25*, *KRT39*, *MAPK12*, *SPP1*, and *TCHHL1*—were identified as regulators of the secondary hair follicle cycle. Specifically, platelet-derived growth factor A (PDGFA), a component of the relaxin signaling pathway, has been shown to promote hair follicle stem cell activation during the catagen-to-anagen transition in cashmere goats [22].

The cAMP signaling pathway is a key pathway that promotes the maintenance of the anagen phase and the activity of hair follicle stem cells in cashmere goat secondary hair follicles, playing a vital role in the positive regulation of the hair follicle cycle, which is consistent with previously reported functions of the cAMP pathway [23,24]. Analysis of 24 genes enriched in this pathway revealed that *HCN4* and *ADCY8* were highly expressed in the telogen phase, and *CRHR2* and *CREB3L1* were highly expressed in the anagen phase, whereas *FOS* showed high expression in the catagen phase. Protein–protein interaction network analysis showed direct connections among certain proteins, with *EDN2* located at the center of the network, linking three protein clusters, and a tight interaction was observed between *CRHR1* and *CRHR2*. This finding is consistent with previous reports that *EDN2* and the *CRHR* family play central roles in signal transduction [13]. Additionally, expression trend analysis of selected genes indicated that *PPP1R1B*, *VIPR2*, and *GRIN1* exhibited consistent expression patterns and were markedly differentially expressed between the anagen and catagen phases, suggesting that they are key effectors mediating the functional transition of the cAMP signaling pathway, which agrees with previously identified cAMP pathway effectors in cycle transition [17]. KEGG enrichment analysis further confirmed that the cAMP signaling pathway acts synergistically with pathways such as PI3K-Akt and MAPK to jointly regulate the proliferation and differentiation of hair follicle stem cells [8,24]. Moreover, the establishment of an in vitro culture system for hair matrix cells, which are the precursor cells for hair shaft formation, provides an important cellular model for studying the hair follicle cycle [25]. The above results verify the positive regulatory role of the cAMP signaling pathway on the anagen phase of cashmere goats’ secondary hair follicles at both the gene expression and protein–protein interaction levels, which is highly consistent with previous research conclusions.

The estrogen signaling pathway may play a negative regulatory role in the initiation of the catagen phase of secondary hair follicles in cashmere goats. Expression profiling of genes enriched in this pathway showed that *PGR* and *ADCY8* were highly expressed in the telogen phase, and *KRT24* and *CREB3L1* were highly expressed in the anagen phase, whereas *FOS* was upregulated again in the catagen phase. Protein–protein interaction network analysis revealed that *KRT27* was located at a core node of the network, forming a regulatory module by connecting keratin family members such as *KRT39* and *KRT26*. *KRT26*, *KRT27*, and *KRT35* exhibited a sustained decreasing trend in expression from the anagen to the catagen phase, with all reaching their lowest levels in the catagen phase. Among these, *KRT26* and *KRT35* were significantly enriched in the estrogen signaling pathway, and their downregulation may be associated with the transition of hair follicles from the anagen to the catagen phase [13]. Furthermore, *KRT26* and *KRT35* have also been suggested to play a role in hair follicle development and fiber formation [26,27]. At the mechanistic level, studies have indicated that estrogen receptors are expressed in both the dermal papilla and stem cells of hair follicles, and estrogen signaling may participate in inducing hair follicles to enter the catagen phase by affecting the activity of hair follicle stem cells [23]. The importance of the estrogen signaling pathway in regulating the hair follicle cycle is further highlighted, and the coordinated downregulation of keratin family members such as *KRT27* and *KRT26*, as potential downstream effector molecules of this pathway, may be involved in the regulatory process governing the transition from the anagen to the catagen phase.

Five key genes potentially regulating the secondary hair follicle cycle were identified: *KRT25*, *KRT39*, *SPP1*, *TCHHL1*, and *MAPK12.* Among them, *KRT25* exhibited the highest expression in catagen. Previous studies reported that *KRT25* is localized to the inner root sheath of hair follicles, suggesting a potential role in maintaining inner root sheath structure [28]. *KRT39* was expressed at significantly higher levels in both anagen and catagen than in telogen; it was highly expressed in long-hair-type individuals and positively correlated with cashmere fiber diameter, suggesting its participation in hair shaft elongation and fiber quality formation [29]. *SPP1* showed high expression in anagen and catagen but extremely low expression in telogen. Located downstream of the PI3K-Akt pathway, *SPP1* may participate in maintaining the hair follicle stem cell niche by regulating extracellular matrix remodeling [30]. Similarly, platelet-derived growth factor A (PDGFA) was highly expressed during the catagen-to-anagen transition and may activate hair follicle stem cells through an autocrine mechanism [30]. *TCHHL1* displayed the highest expression in anagen, intermediate expression in catagen, and the lowest expression in telogen. The phase-dependent expression of *TCHHL1* implies its involvement in the dynamic remodeling of keratin filaments and the extracellular microenvironment, particularly during telogen when the follicle is quiescent and structural reorganization occurs. Notably, the T615C polymorphism of *TCHHL1* has been linked to cashmere fineness, with the CC genotype associated with superior fineness. Taken together, the spatiotemporal expression pattern and the allelic effect on fiber diameter suggest that *TCHHL1* may exert a dual role: modulating hair cycle progression through differential expression and influencing final fiber traits through genetic variation [31]. *MAPK12* was specifically upregulated in telogen and participates in the cascade amplification of cell proliferation signals in synergy with the cAMP pathway [12]. Collectively, the stage-specific expression patterns of these five genes, together with their enrichment in or association with the cAMP and estrogen signaling pathways, suggest that they may be involved in different phases of the secondary hair follicle cycle. However, these transcriptomic associations do not demonstrate direct regulatory functions, and further functional validation is required [30].

## 5. Conclusions

Based on the analysis of skin transcriptome data from Inner Mongolian cashmere goats, a total of 1232 differentially expressed genes (DEGs) associated with the secondary hair follicle cycle were identified. KEGG enrichment analysis revealed that the cAMP, relaxin, and estrogen signaling pathways may be involved in the regulation of hair follicles. GO annotation indicated that the DEGs were mainly enriched in biological processes and functions associated with extracellular space and calcium ion binding. Based on their stage-specific expression patterns, *KRT39*, *MAPK12*, *KRT25*, *SPP1*, and *TCHHL1* were identified as candidate stage-associated genes potentially involved in the secondary hair follicle cycle. These genes provide potential molecular targets for further investigation of secondary hair follicle cycling in cashmere goats; however, their functional roles require further validation through independent samples and experimental approaches.

## Figures and Tables

**Table 1:** SRA data download information.

BioProject	Breed	Tissue	Number	Sample Collection Protocols and Experimental Designs
PRJNA470971	Inner Mongolian cashmere goat	skin	36	Transcriptome data from the skin of three female Inner Mongolian cashmere goats over 12 consecutive months.
PRJNA382893	Inner Mongolian cashmere goat	skin	15	Skin tissue transcriptome data from three female Inner Mongolian cashmere goats in May, June, August, September, and October.
PRJNA592400	Inner Mongolian cashmere goat	skin	9	Skin tissue transcriptome data from three 2-year-old female Inner Mongolian cashmere goats during the anagen (September), catagen (December), and telogen (March) stages of the secondary hair follicle cycle.
PRJNA832904	Inner Mongolian cashmere goat	skin	72	Skin transcriptome data from long-hair and short-hair types of 2-year-old female Inner Mongolian cashmere goats over 12 consecutive months.

**Table 2:** qRT-PCR primers for key genes in the secondary hair follicle cycle.

Gene	Prime	Base Pair	Temperature
*KRT25*	F: AAGTCAGGACCGAGACCGAG	129 bp	61 °C
	R: CAGACTTGCAAGCCCCATCAT		
*KRT39*	F: TGAGATTGCCACATACCGCA	169 bp	59 °C
	R: CTCATGTATCCCACAGGGGC		
*TCHHL1*	F: GCTAAAGGTCCAGAGCCCAA	121 bp	60 °C
	R: TGTCGCAGGGTGTGTCTTTT		
*SPP1*	F: TGAAAGCCCTGAGCAAACAGA	187 bp	60 °C
	R: AGGTGGAGTGAAAACTGCGA		
*MAPK12*	F: CCCGACGAGACACTGGATGA	194 bp	61 °C
	R: AGGTTGCTGGGCTTCAGGT		
*β-actin*	F: GGCAGGTCATCACCATCGG	158 bp	60 °C
	R: CGTGTTGGCGTAGAGGTCTTT		
*GAPDH*	F: GCAAGTTCCACGGCACAG	118 bp	60 °C
	R: TCAGCACCAGCATCACCC		

**Table 3:** Difference analysis table among different secondary hair follicle cycles.

Comparison Group	*P-adj*	log_2_FoldChange	Up	Down	Diff
anagen–catagen	0.01	1	157	58	215
catagen–telogen	0.01	1	139	216	355
telogen–anagen	0.01	1	611	401	1012

**Table 4:** FPKM data table of key genes during the secondary hair follicle cycle.

Gene Name	Anagen	Catagen	Telogen	KEGG
*KRT18*	13.42 ± 12.46	7.55 ± 5.79	22.27 ± 22.77	Estrogen signaling pathway
*KRT24*	0.32 ± 0.42	0.18 ± 0.12	0.05 ± 0.05	Estrogen signaling pathway
*KRT25*	1668.38 ± 937.36	1834.94 ± 556.65	846.67 ± 592.21	Estrogen signaling pathway
*KRT26*	59.24 ± 39.85	64.31 ± 14.57	21.92 ± 17.14	Estrogen signaling pathway
*KRT27*	1320.05 ± 691.70	1417.87 ± 397.72	654.48 ± 450.67	Estrogen signaling pathway
*KRT35*	339.66 ± 141.80	337.35 ± 85.25	160.34 ± 105.59	Estrogen signaling pathway
*KRT38*	22.58 ± 30.06	2.54 ± 1.13	1.85 ± 1.97	Estrogen signaling pathway
*KRT39*	24.42 ± 18.41	19.81 ± 6.99	9.46 ± 6.93	Estrogen signaling pathway
*KRT40*	30.62 ± 14.26	23.50 ± 5.12	12.20 ± 7.24	Estrogen signaling pathway
*CREB3L1*	16.07 ± 9.80	7.98 ± 2.77	11.10 ± 3.86	Estrogen signaling pathway
*CREB3L4*	5.40 ± 2.82	2.72 ± 0.98	6.11 ± 2.56	Estrogen signaling pathway
*FOS*	16.87 ± 16.23	45.03 ± 53.70	19.61 ± 31.13	Estrogen signaling pathway
*ADCY8*	0.11 ± 0.09	0.09 ± 0.08	0.20 ± 0.33	Estrogen signaling pathway
*GREB1*	0.21 ± 0.22	0.21 ± 0.16	0.84 ± 0.34	Estrogen signaling pathway
*CREB3L4*	5.40 ± 2.82	2.72 ± 0.98	6.11 ± 2.56	Estrogen signaling pathway
*CALML6*	0.17 ± 0.14	0.13 ± 0.11	0.28 ± 0.24	cAMP signaling pathway
*CREB3L1*	16.07 ± 9.80	7.98 ± 2.77	11.10 ± 3.86	cAMP signaling pathway
*ABCC4*	4.92 ± 2.50	2.54 ± 0.77	3.02 ± 1.81	cAMP signaling pathway
*PPP1R1B*	3.15 ± 2.13	4.76 ± 3.10	8.27 ± 6.20	cAMP signaling pathway
*GRIN2A*	0.01 ± 0.01	0.01 ± 0.01	0.02 ± 0.03	cAMP signaling pathway
*GRIA3*	1.45 ± 0.75	0.98 ± 0.28	0.62 ± 0.29	cAMP signaling pathway
*MAPK12*	21.02 ± 12.50	24.74 ± 8.53	42.42 ± 15.03	Relaxin signaling pathway
*PTCH1*	7.72 ± 3.41	6.99 ± 1.74	3.59 ± 1.31	Relaxin signaling pathway
*TCHHL1*	37.58 ± 28.71	30.88 ± 19.81	15.34 ± 18.32	Cornified envelope formation
*SPP1*	95.58 ± 61.07	78.05 ± 27.40	37.18 ± 22.69	ECM–receptor interaction

## Data Availability

RNA-Seq data are from PRJNA470971, PRJNA382893, PRJNA592400, and PRJNA832904. The FPKM data of differentially expressed genes are shown in Supplemental Table S3. Please contact the corresponding author for other data (G.G.: ggao1995@163.com).

## Supplementary Materials

The following supporting information can be downloaded at https://www.mdpi.com/article/10.3390/ani16142156/s1, Supplemental Table S1: SRA data download statistics table; Supplemental Table S2: Mapping alignment summary of samples; Supplemental Table S3: FPKM data for all differentially expressed genes; Supplemental Table S4: GO analysis table of differentially expressed genes; Supplemental Table S5: KEGG enrichment analysis table of differentially expressed genes. Figure S1: PCA plot of transcriptome samples showing sample clustering.
