# Supplementary figures and images for: Exploring the Cyclic Patterns of Secondary Hair Follicles in Cashmere Goats Based on Skin Transcriptome Data
Source: Animals (Basel). 2026 Jul 11;16(14):2156. doi: 10.3390/ani16142156 (PMC13403511; doi:10.3390/ani16142156)

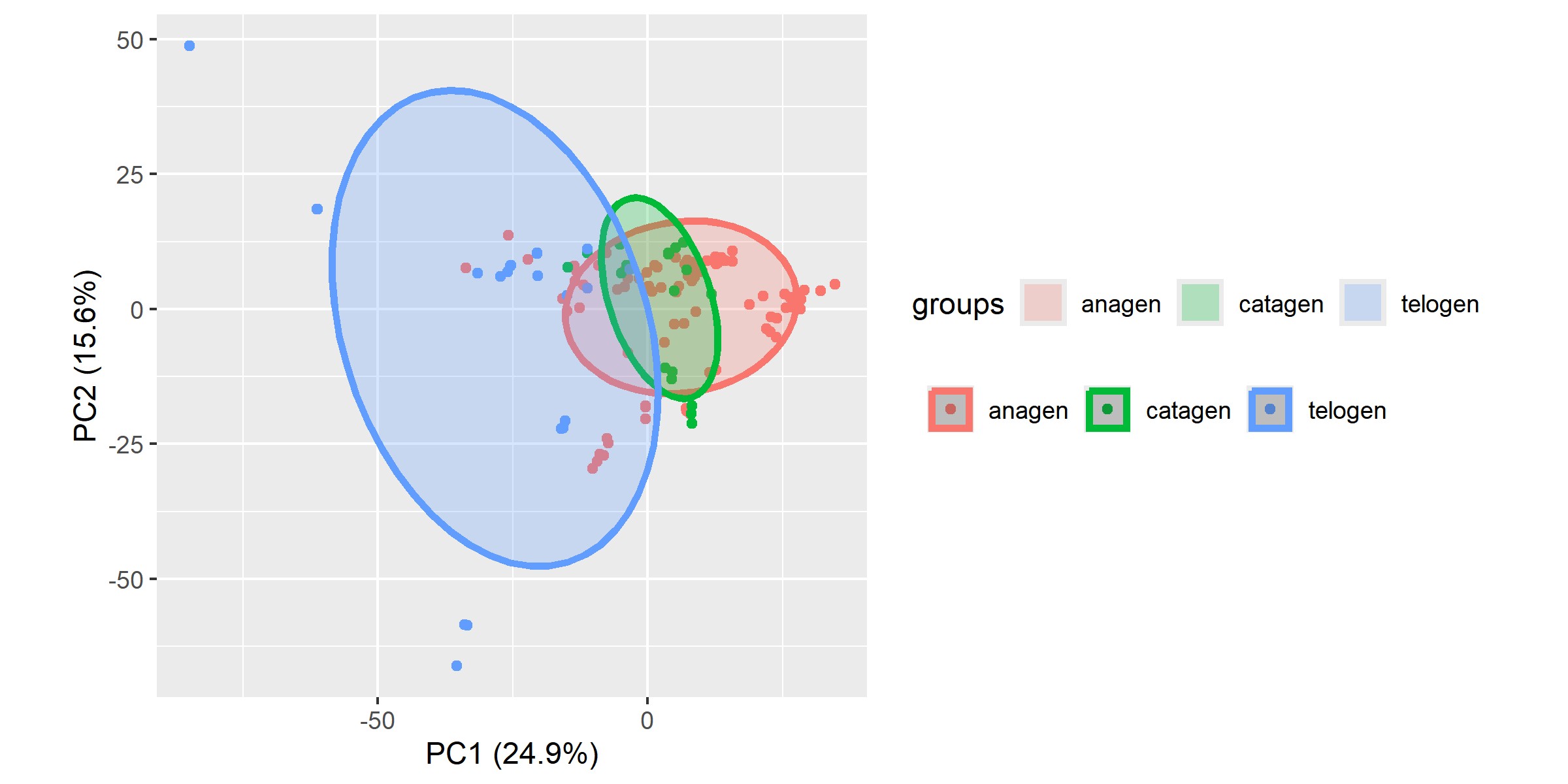

Supplement: Supplementary file 1 [file animals-16-02156-s001.zip › Supplemental Figure S1.jpg]
